# Supplementary figures and images for: An experimental investigation on the dark side of emotions and its aftereffects
Source: PLoS One. 2022 Oct 6;17(10):e0274284. doi: 10.1371/journal.pone.0274284 (PMC9536566; doi:10.1371/journal.pone.0274284)

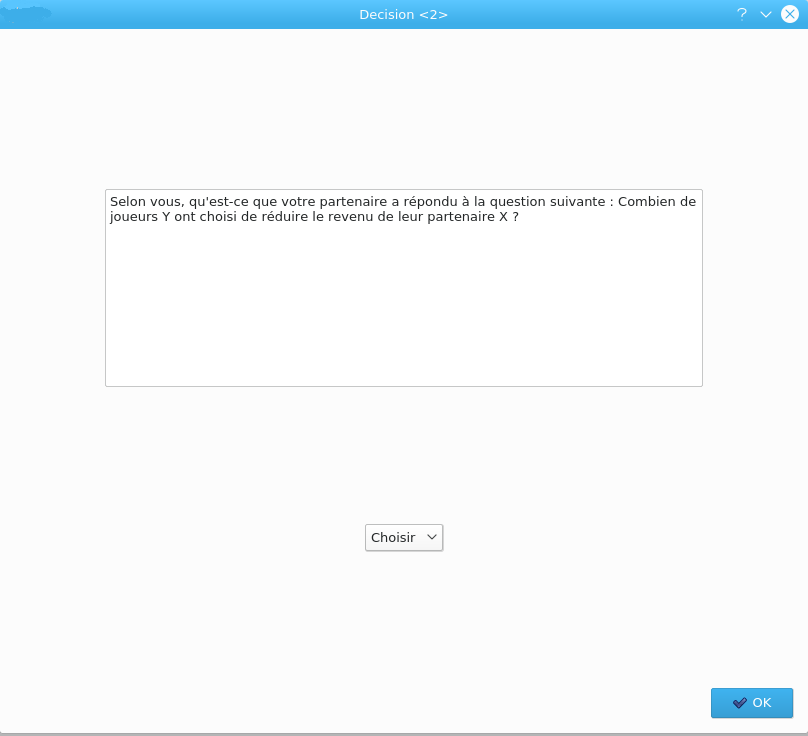

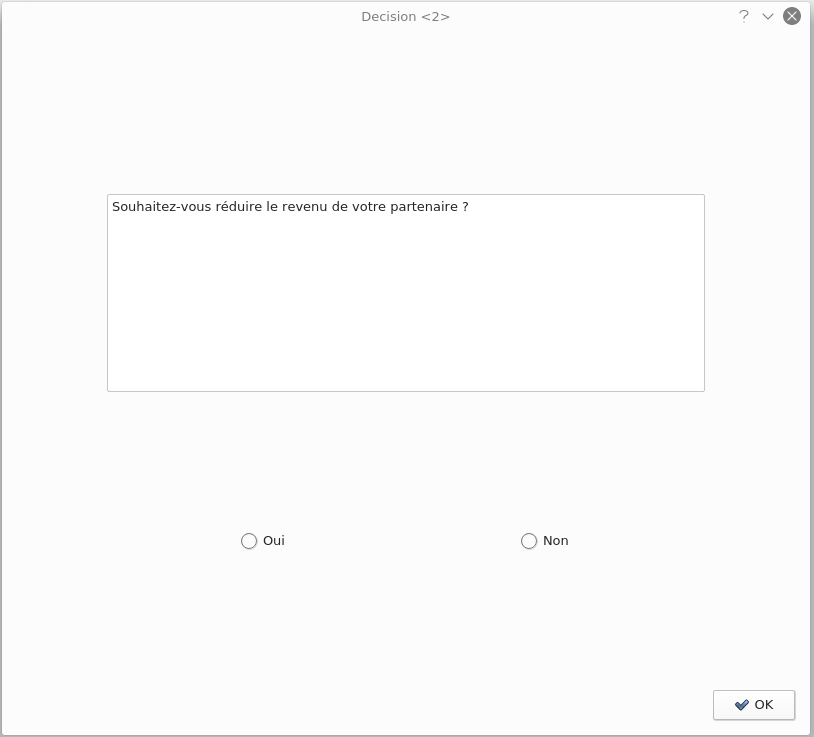

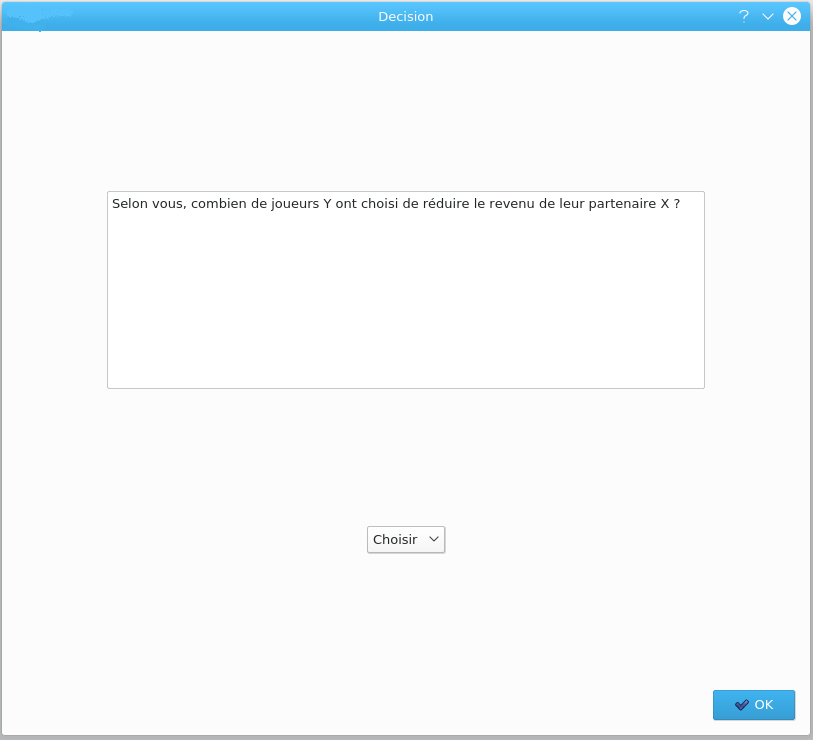

Supplement: S1 File — (DOCX) [file pone.0274284.s001.docx]
