# Supplementary material for: An experimental investigation on the dark side of emotions and its aftereffects
Source: PLoS One. 2022 Oct 6;17(10):e0274284. doi: 10.1371/journal.pone.0274284 (PMC9536566; doi:10.1371/journal.pone.0274284)
Supplement: S2 File — (DOCX) [file pone.0274284.s002.docx]

**Supplementary Materials**

1. *Complete instructions (translated from French)*

The experiment you are going to participate in is for the study of decision making. We ask you to read the instructions carefully, they must allow you to understand the experiment. Once you have read these instructions, an examiner will read them aloud.

All your answers will be treated anonymously throughout the entire experiment. You will indicate your choices thanks to the computer in front of which you are sitting.

The experiment has four parts. At each step, the screen will indicate when you will be able to perform the task or whether to wait. Waiting intervals are sometimes introduced so that everyone involved in the experiment can progress at the same pace.

Your remuneration will be paid in cash at the end of the experiment. Your gain will be randomly determined at the end of the experiment, and corresponds to either the gain of Part 1, the gain of Part 2, or the gain of Part 3.

In all three parts the gains are expressed in ECU. The conversion rate of ECUs in euros is 1 ECU = 15 cent.

We ask you throughout the experiment to remain totally silent and to show no signs that could influence your neighbors. Your laptops should be turned off. You should not view any other documents than those that have been distributed to you or are presented to you on the computer screen.

If you have a question, raise your hand and an experimenter will come to answer you in private.

***PART 1***

The first part consists of two sections.

**Section 1**

The first section is composed of three tasks.

***Task 1***

We ask you to evaluate your level of pleasure as well as your waking state. For this, you have a cursor to move horizontally to express how you feel best.


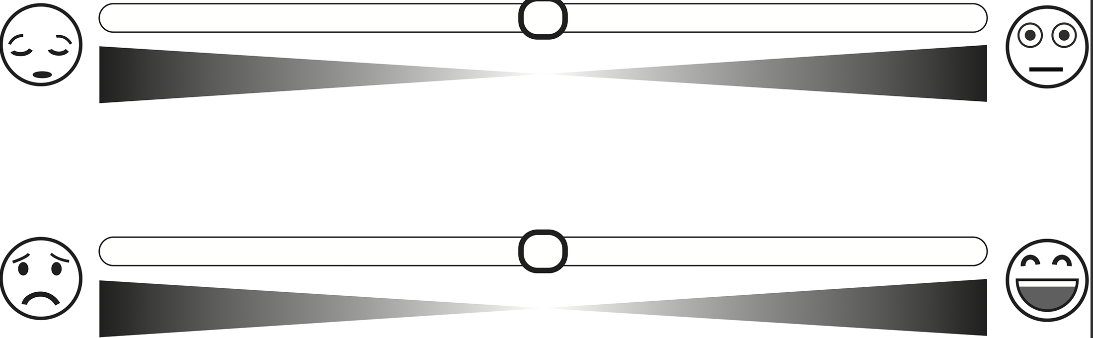


***Task 2***

Photos will be presented. The photos will be the same for all participants. We ask you to watch them carefully.

Then write three adjectives that best describe the feeling felt when viewing photos.

***Task 3***

We ask you to evaluate your level of pleasure as well as your waking state. For this, you have a cursor to move horizontally to express how you feel best.


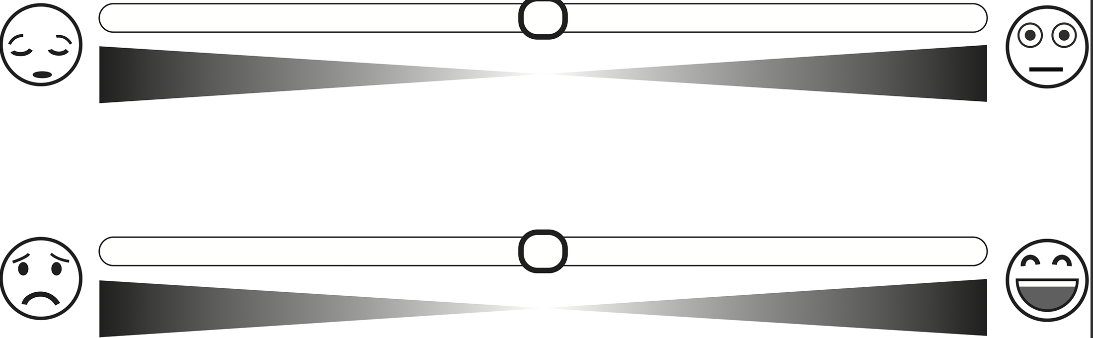


**Section 2**

In this second section, you are randomly paired with another participant - your partner.

You will not learn the identity of the participant with whom you are matched, and vice versa your partner will never know your identity. For each pair, there will be a player X and a player Y.

You will be designated either as player X or as player Y. This will be indicated on your screen before making your decisions.

In the hall there are therefore 10 X players and 10 Y players. Each player receives an endowment of 100 ECU.

We ask you to make three decisions.

***Decision 1***

You and your partner each receive an endowment of 100 ECU.

You have to decide if you want to reduce your partner's income or if you want to leave it unchanged. Reducing your partner's income will cost you 10 ECU.

By paying 10 ECU you can reduce his income by 50 ECU. Your partner makes the same decision at the same time. He can choose between leaving your income unchanged, or reducing it by 50 ECU. Your partner will pay the same cost - 10 ECU - if he chooses to reduce your income.

If you and your partner choose not to change the other person's income, you will earn both 100 ECUs.

If you both choose to reduce each other's income, you will each earn 40 ECU (100 - 50 - 10).

If you choose to reduce the income of your partner, but he / she decides to leave your income unchanged, you will earn 90 ECU and your partner will earn 50 ECU.

If you choose not to reduce the income of your partner, but he / she decides to reduce your income, you earn 50 ECU and your partner will earn 90 ECU.

***Decision 2***

In decision 1, all participants made a decision whether or not to reduce their partner's income. We ask to guess their choices.

If you are an X player, we ask you to guess the number of Y players (including your partner) who have chosen to reduce their partner's income.

If you are a Y player, we ask you to guess the number of X players (including your partner) who have chosen to reduce their partner's income.

If your answer is correct, i.e., if your answer corresponds to the exact realization, you gain 20 additional ECUs.

So, for example, if you are a player X and you indicate that 5 players Y have reduced the income of their partner, and that there are really 5 players Y who made this choice, you gain 20 ECU. On the other hand, if only 3 Y players have really reduced their partner's income, you will not win anything.

***Decision 3***

In decision 2, your partner also guessed the number of players (having the same role as you) who chose to reduce their partner's income in decision 1. We ask you to indicate what you think was your partner's answer.

If your answer corresponds to the real decision of your partner (decision 2 of your partner), you gain 20 additional ECUs.

So if you indicate that your partner has answered that 5 players (having the same role as you) have reduced the income of their partner, and that actually 5 players have made this choice, you earn 20 ECU. On the other hand, if only 3 players have really reduced the income of their partner, you do not win anything.

***PART 2***

In this part of the experiment, you are randomly paired with another participant - your partner. You will not learn the identity of the participant with whom you are paired, and vice versa your partner will never know your identity. This is a new toss, so your partner in this second part of the experiment is not necessarily the partner in the first part of the experimeent.

You have 6 decisions to make. For the 6 decisions, we ask you to make your choice as player A, by using a cursor which will determine the gains for both players A and B.

At the end of the experiment, if this part is drawn for payment, the computer will randomly draw one of the 6 decisions for actual payment.

Your gain of this game is determined as follows. A coin toss determines your role (ie whether you are player A or player B).

In the event that the toss decides that you are player A, your gain is determined by your actual decision. Your gain will be the amount you have decided to award to player A. In this case, your partner will win the amount you have awarded to player B.

In the event that the toss decides that you are player B, your gain depends on your partner's decision. In this case, you will win the amount that your partner has decided to award to player B.

***PART 3***

This third part of the experiment is made up of two tasks. For each task, you have 20 decisions to make. If this part of the experiment is drawn for payment, the computer will randomly draw one of the 40 decisions and determine your gain in the case this part of the experiment is randomly chosen for your final payment.

***Task 1***

You have 20 decisions to make. For the 20 decisions, you are invited to choose between option A and B.

If you choose option A, you will receive a certain amount of ECU.

If you choose option B, the computer will randomly choose one of 10 balls from an urn. Each ball is either blue or yellow: if the color of the ball chosen by the computer is yellow, you will earn 150 ECU, if the ball is blue you will earn 0 ECU.

**Among the 10 balls, there are 5 blue balls and 5 yellow balls.**

***Task 2***

In this part of the experiment, you have 20 decisions to make. For the 20 decisions, you are invited to choose between option A and B.

If you choose option A, you will receive a certain amount of ECU.

If you choose option B, the computer will randomly choose one of 10 balls from an urn. Each ball is either blue or yellow: if the color of the ball chosen by the computer is yellow, you will earn 150 ECU, if the ball is blue you will earn 0 ECU.

**You don't know how many yellow balls are among the 10 balls. Any composition of the two colors is possible.**

***Final questionnaire***

We ask you to answer a series of questions. For each question, you must indicate whether you strongly agree, agree, neither agree nor disagree, disagree, or strongly disagree.

Please answer the questions as sincerely as possible. There are no right or wrong answers.

| \|  \| **Disagree strongly** \| **Disagree** \| **Neither agree nor disagree** \| **Agree** \| **Agree strongly** \| \| --- \| --- \| --- \| --- \| --- \| --- \| \| It’s not wise to tell your secrets \| □ \| □ \| □ \| □ \| □ \| \| I like to use clever manipulation to get my way \| □ \| □ \| □ \| □ \| □ \| \| Whatever it takes, you must get the important people on your side \| □ \| □ \| □ \| □ \| □ \| \| Avoid direct conflict with others because they may be useful in the future \| □ \| □ \| □ \| □ \| □ \| \| It’s wise to keep track of information that you can use against people later. \| □ \| □ \| □ \| □ \| □ \| \| You should wait for the right time to get back at people \| □ \| □ \| □ \| □ \| □ \| \| There are things you should hide from other people to preserve your reputation \| □ \| □ \| □ \| □ \| □ \| \| Make sure your plans benefit yourself, not others \| □ \| □ \| □ \| □ \| □ \| \| Most people can be manipulated \| □ \| □ \| □ \| □ \| □ \| |
| --- | --- | --- | --- | --- | --- | --- | --- | --- | --- | --- | --- | --- | --- | --- | --- | --- | --- | --- | --- | --- | --- | --- | --- | --- | --- | --- | --- | --- | --- | --- | --- | --- | --- | --- | --- | --- | --- | --- | --- | --- | --- | --- | --- | --- | --- | --- | --- | --- | --- | --- | --- | --- | --- | --- | --- | --- | --- | --- | --- | --- |
