# Supplementary material for: An experimental investigation on the dark side of emotions and its aftereffects
Source: PLoS One. 2022 Oct 6;17(10):e0274284. doi: 10.1371/journal.pone.0274284 (PMC9536566; doi:10.1371/journal.pone.0274284)
Supplement: Attachment — Submitted filename: Review for article PONE D 21 35528.pdf [file pone.0274284.s004.pdf]

Review for article PONE D 21 35528

An experimental investigation on the dark side of emotions and its aftereffects

Summary:

This paper aims at investigating the role of beliefs and incidental emotions on destruction behavior in a Joy of Destruction experimental game. The two main determinants of destruction are spite and pre-emptive retaliation (negative reciprocity based on beliefs). It is found that positive incidental emotions have no direct impact on behavior and on beliefs on partners' behavior (1st order beliefs). However, they have a direct impact on beliefs on partners' beliefs (2nd order beliefs), which in turn influences positively 1st order beliefs and eventually behavior. Hence positive incidental emotions indirectly slightly reduce destruction behavior.

Overall assessment

This is an interesting and well-written article. The research question is relevant and original because it deals with (1) bad behavior instead of virtuous behavior, (2) incidental instead of experienced emotions, (3) 2nd order beliefs and not only 1st order beliefs.

I have however important concerns that I would like to be handled by the authors before recommending publication:

- First, the experimental procedures appear to be correct, although **the design should be better justified**. Overall, the links between the theoretical section, the experimental design section and the results section should be clearer.
- Second, **I am not fully convinced by the statistical analysis for now, especially the econometric part**. The models and methods used should be better described and justified. Explanatory variables are not correctly defined. The interpretations of some results are debatable. At the end of the day, it seems to me that the conclusion of the article overstates the results of the experiment: the impact of incidental emotions is perhaps not as strong as seemed to be claimed.

Detailed comments:

Introduction

- Overall, it is well written.
- Typos:
    - Page 6: Strange phrase: "We show that choosing to destroy others' endowment is, at least to some, rational that is based on expectations."

Theoretical framework and behavioral hypotheses

- Page 9: Could Hypothesis 1 be made more precise with respect to the model, stating clearly which variables of the model are involved? In other words, does Hypothesis 1 mean that the probability of destruction increases with P_i and decreases with S_i and α_i^j? Indeed, the term "degree of spitefulness" used in the hypothesis is a bit ambiguous.

- Page 10: Hypothesis 3 is convincing and the justifications are fine. However, Fochmann et al. (that the authors state just before) seem to go in the opposite direction: "Similarly, Fochmann et al. [27] provide evidence that individuals have a lower willingness to comply with social norms after being primed with positive incidental emotions compared with aversive emotions." Arguably, following this argument, one might expect that after positive incidental emotion, subjects might be willing to destroy more? In other words, Hypothesis 3 seems to be intuitive but are there counterarguments in the literature?

- Is it possible to make hypotheses regarding which variables of the model are impacted by incidental emotions? Will the impact be on P_i? S_i? Or on the 1st order belief? Or on the 2nd order belief? That is, are people directly affected by incidental emotions or do they believe that others will be? Maybe it would be relevant to introduce the impact of emotions into the mathematical model?

- Typos:
  - Page 8, top: Maybe this terminology is standard in this literature but to me, S_i should not be referred to as "spitefulness", this is reciprocity (more specifically retaliation). P_i could be referred to as spitefulness.

Experimental design

- The experimental procedures seem to be very good. The protocol allows to collect a variety of data. Nevertheless, this section is very descriptive overall. The authors should not only describe but also briefly justify their design: for example, why elicitating risk preferences? Social value orientation?... What are the conjectures regarding these variables? For example, is a more risk-averse subject expected to destroy more? (this is what I would say intuitively: in the absence of destruction, the variance of gains is large, whereas destructing is like a costly insurance as it provides a "revenge utility" which partly compensates the potential harm of the other player) And why using these specific procedures instead of others? (e.g., why this risk aversion test and not Holt-Laury?) For sure, the text should however be kept concise.

- Typos:
  - Page 12: I guess that the social value orientation game is Part 2 in the instructions? If so, the sequence presented in Table 1 does not seem to match exactly with the sequence presented in the instructions.

Results

- Table 2: aren't the authors also interested in the elicited level of arousal (as well as valence)?
- The treatment comparison tables are not very easy to read. For example, in Table 3, what is exactly the "-0.467"? It is possible to guess after some thought, but authors might be willing to help the reader in notes below the tables. Figures 2 and 3 are also not very clear (what are the red curves?). In Table 4, I do not understand exactly what the "4.430" and "3.613" are; I would expect them to be negative (?).
- Page 16: I'm not convinced by the way Result 2 is written: "Positive incidental emotions lower individual's expectations of other players' destructive behavior and beliefs." → You should mention that it is relative to the neutral emotion since no significant difference with the negative emotion treatment shows up. It is surprising that negative emotions have no significant impact, and if anything, that they tend to curb decisions and beliefs in the same direction as positive emotions. Would the authors have explanations for this?
- Overall, the econometric analysis may be more detailed. For example, are you using specific econometric methods? What are the assumptions regarding the variance-covariance matrix of the error term? Why a Tobit regression? How many left- and right censored values are there? The explanatory variables are not defined (what is the definition of variable "gender" for example? What is "risk aversion"? What about the measure of ambiguity aversion?) and descriptive statistics on these variables should be provided. Table 5, model (2): it seems to me that getting a pseudo-R^2 larger than 1 reveals that the model fits the data poorly. Could the authors check this?
- Table 5, models (2) and (3): it could be clearer to present first regressions without interaction terms since they complicate interpretations. For example, in model (3), the term "Positive emotion" is significantly negative. But because of the presence of the term "Positive*Destruction", it means that the former should be interpreted as the effect of positive emotions *for the subjects who do not destroy*. It seems actually that the global marginal effect of "Positive emotion" (-0.075+0.0885) is actually very small?
- …Still Table 5: I don't know if it is a good idea to include "Destruction" into models (2) and (3) since it looks like a circular reasoning: beliefs are supposed to be a determinant of destruction, not the opposite. Beware of endogeneity issues. (Same issue with "Expected partner's Behavior" in model (3).)
- …Still Table 5: some results are simply stated without much explanation although they appear to be counterintuitive. For example, "participants who inflict destruction to their partners expect their partners to believe they will adopt less destructive behavior than will expect participants who decide not to destroy the endowment of their partner (Model 3)." Other results are ignored, such as for example the effect of risk aversion. More risk-averse subjects expect their partners to believe they will adopt less destructive behavior. Is it possible to find some explanation for this?
- Structural equation model:
    - The estimation procedure used for the structural equation model should be more detailed. I am wondering whether model (4) would not be sufficient instead of models (1) to (3), since it would avoid circular reasoning as mentioned before.

- Why are there so few explanatory variables in the second regression? It was shown in regression (3) that positive emotion has a negative impact on partner's expectation. So, why not considering a three-equation structural model, regressing 2nd order beliefs on various control variables such as emotion, then regressing 1st order beliefs on the 2nd order beliefs and on same set or other control variables, then regressing willingness to destroy 1st order beliefs and on the same set of variables or others? The first regression should allow to show that emotions impact significantly 2nd order beliefs.

- Typos:
    - Page 18-19: The sentence "Participants who have already participated in lab experiments have lower expectations on their partner's willingness to destroy." is written twice.

Discussion & conclusion

- The main result is interesting: incidental emotions would not impact destruction behavior directly, or through 1st order beliefs, but only 2nd order beliefs. Are there other studies on the impact of emotions on 2nd order beliefs? Are there some psychological or neurological explanations or hypotheses regarding this phenomenon?
- The conclusions should be qualified given Result 1: there seems to be an effect of incidental emotion, but this effect appears to be small anyway. Overall, the conclusion seems to slightly overstate the results of the paper. For example, the phrase "Indeed, positive emotions induce people to be more optimistic on others' behavior and beliefs and thus reduces preemptive retaliation" is correct based on the Mann-Whitney comparison tests but appears to be less clear from the econometric analysis (i.e., after controlling for various factors).
- Typos:
    - The first sentence of the conclusion is strangely written.
    - Page 22: "we deduced that individuals in whom positive emotions were induced TO destroy less than others."

Instructions

- Typo: "Experiment" instead of "experience".
