# Supplementary material for: An experimental investigation on the dark side of emotions and its aftereffects
Source: PLoS One. 2022 Oct 6;17(10):e0274284. doi: 10.1371/journal.pone.0274284 (PMC9536566; doi:10.1371/journal.pone.0274284)
Supplement: Attachment — Submitted filename: Review for article PONE D 21 35528_R1.pdf [file pone.0274284.s005.pdf]

Review for article PONE D 21 35528

An experimental investigation on the dark side of emotions and its aftereffects

Summary:

This paper aims at investigating the role of beliefs and incidental emotions on destruction behavior in a Joy of Destruction experimental game. The two main determinants of destruction are spite and pre-emptive retaliation (negative reciprocity based on beliefs). It is found that positive incidental emotions have no direct impact on behavior and on beliefs on partners' behavior (1st order beliefs). However, they have a direct impact on beliefs on partners' beliefs (2nd order beliefs), which in turn influences positively 1st order beliefs and eventually behavior. Hence positive incidental emotions indirectly reduce destruction behavior.

Overall assessment

I think that the authors did great job in improving the paper. I have still a lot of comments, most of which can now be considered as minor, **except maybe the two comments that are presented first below, regarding subtle effects of positive incidental emotions on behavior and beliefs**: I think you need to 1) better estimate them, 2) propose an interpretation for them.

Detailed comments:

Main issues

- **Page 22, "Result 4: Positive emotions reduce destruction for individuals with low traits of Machiavellianism."** → I think the support for this result should be made stronger. You derive it from the results of the regression done in model 4 (table 6b). You state: "Indeed, the variable Positive emotions as well as the interaction term between Positive emotions and Machiavellianism being significant, means that for individuals with low Machiavellian scores, positive emotions lower their willingness to destroy partner's endowment, whereas for people with high scores destruction increases." However, I think you should compute proper marginal effects to reach these conclusions. For example, compute

  $$Prob(Destruction = 1|\ Positive\ emotions = 1, Machiavellism = 1) - Prob(Destruction = 1|\ Positive\ emotions = 0, Machiavellism = 1)$$

  to properly estimate the effect of positive emotions for low Machiavellian subjects. You will probably confirm Result 4. I would be curious to know the effect for highly Machiavellian subjects

  $$Prob(Destruction = 1|Positive\ emotions = 1, Machiavellism = 5) - Prob(Destruction = 1|Positive\ emotions = 0, Machiavellism = 5),$$

which is maybe significantly positive, meaning that highly Machiavellian subjects tend to destruct more under positive emotions. If so, why? It is not intuitive that positive emotions should lead to more destruction for this type of subjects.

- **Page 23: I have similar comments as above.** On the basis of the regression of model 6, you state: "Regarding the impact of emotion, we observe that positive emotions moderate expectations on destruction according expectations on partners' belief. For individuals with low expectations on partners' beliefs, positive emotions lower their expectations on partners' destructive behavior, whereas for individuals with high expectations on partners' beliefs, expectations that partners decide to destroy increases." → Again, you need to prove this by computing proper marginal effects. Again, would you have an interpretation? It is not intuitive that positive emotions should lead expected partner's destruction to increase.

Minor issues

Introduction

- Page 2, second parag.: no comma after "While".

Theoretical framework and behavioral hypotheses

- Page 6: "The reciprocity utility has two components: S_i which correspond to individual i's satisfaction of destruction or spitefulness and $R_i \alpha_i^j$ the reciprocation term which..." → No, the reciprocity utility does not include S_i.
- Page 10: "Hypothesis 3: Participants' second-order beliefs influence destruction through first-order beliefs." → You could even state that the relation is expected to be increasing, right?

Experimental design

- Page 10: Regarding the power analysis. This is a good point but it seems to me that your statement is too imprecise. Power analysis requires that you specify the effect size that you are expecting.
- Page 12: "We suppose second order beliefs to be a proxy for R, i.e., player's willingness to reciprocate." → This sentence is misleading, why not: "We suppose second order beliefs to be a proxy for R, i.e., *the other* player's willingness to reciprocate." However, even though I see what you mean, I am a bit confused with this interpretation which seems to come from nowhere. Why not presenting and discussing it properly in the theoretical section (if it is worth it)? In the model, beliefs (the alpha's) and the willingness to reciprocate (the R's) are separate parameters so this assumption is not so obvious.
- Page 14, table 2: Regarding the variables such as Expected partner's behavior, wouldn't they be more informative in relative terms? Besides, how many subjects are there in a session (I guess 10 from the following, but it seems that you never mention it explicitly). In addition, check the definition of Expected partner's belief in the table. It seems to be wrong.

Results

- Page 15:
    - "We start by examining…" → This paragraph is not clearly structured. You should explain that it is more relevant here to run diff. in diff. tests than simple diff. tests. This is not so obvious at first sight.
    - In your answers to my previous comments, you seemed to state that you used Logit and Tobit regressions to make up for non-normal error terms. However, these are still parametric analyses (thus, based on normality), so that I don't think this is the correct justification. You are using Logit regressions because the dependent variable is binary and a Tobit regression because the dependent variable is left/right censored (specify the number of censored observations).
- Table 3: Please specify the number of observations.
- Page 17: "Then, the hypothesis that the proportion of individuals who destroy another player's money is significantly different under the "positive", "negative" and "neutral" emotional state is rejected at the 10% level." → The sentence seems to be wrongly stated, better: "The hypothesis of no difference across treatments cannot be rejected at 10%."
- Page 18: the p=0.0584 does not match with the one in the table.
- Page 19: "an increase of expectation (i.e. one extra partner out of ten) increases the probability (between 0 and 1) to adopt destructive behavior with 0.045." → You mean "a unitary increase in expectation". So, it means that a 10% increase in expectation leads to a 4.5% increase in destruction? right? Maybe it would be more conventional to state it as an elasticity (thus, for a 1% increase). Moreover, can you interpret this? Is it rather inelastic? In a similar vein, check the next sentence as well.
- Page 21, top: "Both results are in line with Hypothesis 1." → Why don't you mention that "Expected partners' beliefs" is not significant, which contradicts H1? Apparently, you drop this variable in table 6b but you should explain why. Besides, table 6b is not really introduced in the text. The transition between table 6a and 6b is a bit abrupt.
- Page 22:
    - One more time, why using a Tobit regression? You could justify it by specifying the number of left/righ censored observations.
    - Typo : "and then regreSS first order beliefs on the second order beliefs (Table 7). To do so, we use censored Tobit regressions…"
- Page 23: "we should test if there exists an indirect effect of expectations about others' beliefs on destruction" → the end of the paragraph is strange. You seem to say that you are now going to show that there is an indirect effect, but this is what you just showed in table 7. So, instead of writing "we should test if there exists an indirect effect of expectations about others' beliefs on destruction. As expected, we found an affirmative response to this assumption.", I would simply

write “This shows that there exists an indirect effect of expectations about others’ beliefs on destruction.”.

- Page 25:
    - "Indeed, experienced participants are closer to real behaviors. " → I think that this is debatable. On the contrary, one might argue that experienced participants have learnt specific behavior that may bias their decisions. For example, they may be used to detect the goal of the experiment. In the positive emotion treatment, they may suspect that the experimenter expects subjects to destroy less, and so be influenced by this conjecture(?)
    - Typo: “and expect that partners belieVE them to be less destructive.”
    - I don’t understand your interpretation regarding the effect of risk aversion. Besides, at the end of the day, even though you collect ambiguity aversion, you never include it in the regressions (you only focus on risk aversion).
    - Another global remark on the regressions: you do not consider arousal in the regressions. Is this because the effects are too small? You can simply state it before presenting the regressions.

Discussion & conclusion

- Page 26:
    - Typo: “we deduce that individuals in whom positive emotions were induced TO destroy less than others. In other words,”
