# Supplementary material for: An experimental investigation on the dark side of emotions and its aftereffects
Source: PLoS One. 2022 Oct 6;17(10):e0274284. doi: 10.1371/journal.pone.0274284 (PMC9536566; doi:10.1371/journal.pone.0274284)
Supplement: Attachment — Submitted filename: Reply_Revision2.docx [file pone.0274284.s006.docx]

**Review for article PONE ID 21 35528**

An experimental investigation on the dark side of emotions and its aftereffects

Dear Editors,

Thank you for giving us the opportunity to submit a revised draft of our manuscript titled “**An experimental investigation on the dark side of emotions and its aftereffects”** to *PLOS ONE*. We appreciate the time and effort that you and the reviewers have dedicated to providing your valuable feedback on our manuscript. We are grateful to the reviewers for their insightful comments on our paper. We have been able to incorporate changes to reflect most of the suggestions provided by the reviewers. We have highlighted the changes within the manuscript.

Here is a point-by-point response to the reviewers’ comments and concerns

**Reviewer 1**

An experimental investigation on the dark side of emotions and its aftereffects

Summary:

This paper aims at investigating the role of beliefs and incidental emotions on destruction behavior in a Joy of Destruction experimental game. The two main determinants of destruction are spite and pre-emptive retaliation (negative reciprocity based on beliefs). It is found that positive incidental emotions have no direct impact on behavior and on beliefs on partners’ behavior (1st order beliefs). However, they have a direct impact on beliefs on partners’ beliefs (2nd order beliefs), which in turn influences positively 1st order beliefs and eventually behavior. Hence positive incidental emotions indirectly reduce destruction

behavior.

Overall assessment

I think that the authors did great job in improving the paper. I have still a lot of comments, most of which can now be considered as minor, except maybe the two comments that are presented first below, regarding subtle effects of positive incidental emotions on behavior and beliefs: I think you need to 1) better estimate them, 2) propose an interpretation for them.

We would like to thank you again for your careful reading, interesting suggestions and support in helping us to improve our paper. For convenience, we reproduce each of your comments below, followed by our responses in blue.

Detailed comments:

**Main issues**

- Page 22, “Result 4: Positive emotions reduce destruction for individuals with low traits of

Machiavellianism.” → I think the support for this result should be made stronger. You derive it from the results of the regression done in model 4 (table 6b). You state: “Indeed, the variable Positive emotions as well as the interaction term between Positive emotions and Machiavellianism being significant, means that for individuals with low Machiavellian scores, positive emotions lower their willingness to destroy partner’s endowment, whereas for people with high scores destruction increases.” However, I think you should compute proper marginal effects to reach these conclusions. For example, compute

𝑃𝑟𝑜𝑏(𝐷𝑒𝑠𝑡𝑟𝑢𝑐𝑡𝑖𝑜𝑛 = 1| 𝑃𝑜𝑠𝑖𝑡𝑖𝑣𝑒 𝑒𝑚𝑜𝑡𝑖𝑜𝑛𝑠 = 1, 𝑀𝑎𝑐ℎ𝑖𝑎𝑣𝑒𝑙𝑙𝑖𝑠𝑚 = 1) −

𝑃𝑟𝑜𝑏(𝐷𝑒𝑠𝑡𝑟𝑢𝑐𝑡𝑖𝑜𝑛 = 1| 𝑃𝑜𝑠𝑖𝑡𝑖𝑣𝑒 𝑒𝑚𝑜𝑡𝑖𝑜𝑛𝑠 = 0, 𝑀𝑎𝑐ℎ𝑖𝑎𝑣𝑒𝑙𝑙𝑖𝑠𝑚 = 1)

to properly estimate the effect of positive emotions for low Machiavellian subjects. You will

probably confirm Result 4. I would be curious to know the effect for highly Machiavellian subjects

𝑃𝑟𝑜𝑏(𝐷𝑒𝑠𝑡𝑟𝑢𝑐𝑡𝑖𝑜𝑛 = 1|𝑃𝑜𝑠𝑖𝑡𝑖𝑣𝑒 𝑒𝑚𝑜𝑡𝑖𝑜𝑛𝑠 = 1, 𝑀𝑎𝑐ℎ𝑖𝑎𝑣𝑒𝑙𝑙𝑖𝑠𝑚 = 5) −

𝑃𝑟𝑜𝑏(𝐷𝑒𝑠𝑡𝑟𝑢𝑐𝑡𝑖𝑜𝑛 = 1|𝑃𝑜𝑠𝑖𝑡𝑖𝑣𝑒 𝑒𝑚𝑜𝑡𝑖𝑜𝑛𝑠 = 0, 𝑀𝑎𝑐ℎ𝑖𝑎𝑣𝑒𝑙𝑙𝑖𝑠𝑚 = 5),

which is maybe significantly positive, meaning that highly Machiavellian subjects tend to destruct more under positive emotions. If so, why? It is not intuitive that positive emotions should lead to more destruction for this type of subjects.

Thank you for pointing this issue. We tried to give better support for our results.

First, we now only indicate marginal effects. Second, the Machiavellian variable is a continuous variable (from 1.33 to 5), so the interpretation of the crossed variable (Machiavellian*Positive emotion) is that the higher the Machiavellian traits the more positive emotions have a significant impact on destructive behavior.

We also tried to better expain the findings. Indeed, it is not to question the beneficial consequences that positive emotions, positive thinking and positive attitudes derive such as, cooperativeness, flexibility, altruism and life satisfaction, etc. (e.g., Schwarz, 2000; Forgas, 2000). Rather, numerous empirical studies have revealed the darker side of happiness. In this vein, Gruber et al. (2011) indicated that experiencing happiness is not always positive. People who are in the pursuit of happiness tend to be to be more depressed, miserable, and unhappy. According to Tan and Forgas (2010) there exists a positive and significant relationship between happiness and selfishness. Further, Tamir and Bigman (2014) demonstrate that positive emotions can have a negative impact on people, and that negative emotions can have a positive impact. Thus, it is assumed that the possibility for positive emotion to have a significant impact on increasing Machiavellian subjects’ destructive behavior cannot be eliminated. Adding to this, previous research has showed that individuals with high levels of Machiavellianism usually endorse ego-centric or antisocial notions of well-being where they tend to prioritize their personal happiness over other people’s happiness (Kajonius et al., 2015). Consequently, it is presumed that these persons have decided to do whatever it takes to keep the positive emotional state even if their actions will have negative consequences or/and violate their own moral values.

- Page 23: I have similar comments as above. On the basis of the regression of model 6, you state:

“Regarding the impact of emotion, we observe that positive emotions moderate expectations on destruction according expectations on partners’ belief. For individuals with low expectations on partners’ beliefs, positive emotions lower their expectations on partners’ destructive behavior, whereas for individuals with high expectations on partners’ beliefs, expectations that partners decide to destroy increases.” → Again, you need to prove this by computing proper marginal effects. Again, would you have an interpretation? It is not intuitive that positive emotions should lead expected partner’s destruction to increase.

Thank you for pointing this issue. We now perfom the econometric analyses properly as expectations for detruction and beliefs are integer variables, and again only report marginal effects.

As explained in the previous point, positive emotions can have a negative impact on individuals’ thoughts and behaviors. It is worthy to mention that a growing body of research investigating the relationship between emotional experience and social information processing indicates that when people are performing a social judgment task the type of strategy they use may be affected by their momentary emotional state (for a review, see for example Bodenhausen, 1993). In the same vein, certain positive emotions lead people to rely more on highly accessible cognitions, such as beliefs, expectations, and stereotypes (e.g., Forgas & Fiedler, 1996). Importantly, happiness has been usually associated with the use of more superficial or cursory styles of thinking. For instance, some studies on mood and persuasion (e.g., Schwarz, Bless, & Bohner, 1991) support this evidence and document that prior to the presentation of a persuasive message, happy people are less affected by variations in argument quality. Indeed, happy people often prefer to base their reactions more on simple cues ( Petty, Gleicher, & Baker, 1991). Thus, we assume that, under a positive emotional state, individuals has adopted a superficial thinking that disabled them from engaging in close scrutiny of the situation. It appears that these individuals expected their partner’s destruction to increase because they has interpreted the suspicious situation as a threat to be handeled with precaution.

**Minor issues**

Introduction

- Page 2, second parag.: no comma after “While”.

Corrected as suggested

Theoretical framework and behavioral hypotheses

- Page 6: “The reciprocity utility has two components: 𝑆𝑖 which correspond to individual i's

satisfaction of destruction or spitefulness and 𝑅𝑖𝛼𝑖𝑗 the reciprocation term which...” → No, the reciprocity utility does not include 𝑆𝑖.

Thank you for this remark, and we agree that S isn’t included in the reciprocity utility. We replaced “the reciprocity utility” by “the non-monetary utility of destruction”.

- Page 10: “Hypothesis 3: Participants’ second-order beliefs influence destruction through first-order beliefs.” → You could even state that the relation is expected to be increasing, right?

Thank you for this interesting suggestion, we modified Hypothesis 3 accordingly.

Experimental design

- Page 10: Regarding the power analysis. This is a good point but it seems to me that your statement is too imprecise. Power analysis requires that you specify the effect size that you are expecting.

Thank you for pointing this issue. We now indicate the expected effect size for our sample. We added the following sentences to the manuscript.

We ran an ex-post power analysis using the G*power software which revealed that our sample size (respectively 55, 57 and 60 observations for the three treatments) is sufficient (power=0.8) to observe an effect size of 0.4. While Cohen (1992) recommends that wherein d = 0.20 indicates a small effect, d = 0.50 indicates a medium effect and d = 0.80 indicates a large effect, Dancey & Reidy (2006) specify that small and medium effects are more willingly available when evaluating behavioral and psychological constructs as it is the case of constructs investigated herein.

- Page 12: "We suppose second order beliefs to be a proxy for 𝑅, i.e., player’s willingness to reciprocate." → This sentence is misleading, why not: "We suppose second order beliefs to be a proxy for 𝑅, i.e., the other player’s willingness to reciprocate." However, even though I see what you mean, I am a bit confused with this interpretation which seems to come from nowhere. Why not presenting and discussing it properly in the theoretical section (if it is worth it)? In the model, beliefs (the alpha’s) and the willingness to reciprocate (the R’s) are separate parameters so this assumption is not so obvious.

Thank for very much for this suggestion. We now present R to be partner’s expectations on the other player’s willingness to reciprocate directly in the theoretical section.

- Page 14, table 2: Regarding the variables such as Expected partner’s behavior, wouldn't they be more informative in relative terms? Besides, how many subjects are there in a session (I guess 10 from the following, but it seems that you never mention it explicitly). In addition, check the definition of Expected partner’s belief in the table. It seems to be wrong.

We have corrected as suggested.

Results

- Page 15:

o “We start by examining...” → This paragraph is not clearly structured. You should explain

that it is more relevant here to run diff. in diff. tests than simple diff. tests. This is not at first sight.

We agree and now discuss both the simple difference as well as the diff in diff tests.

o In your answers to my previous comments, you seemed to state that you used Logit and Tobit regressions to make up for non-normal error terms. However, these are still parametric analyses (thus, based on normality), so that I don’t think this is the correct justification. You are using Logit regressions because the dependent variable is binary and a Tobit regression because the dependent variable is left/right censored (specify the number of censored observations).

Thank you for pointing this out and we absolutely agree with your arguments. We now justify the censored Tobit regression indicating the number of censored observations.

- Table 3: Please specify the number of observations.

Corrected as suggested

- Page 17: “Then, the hypothesis that the proportion of individuals who destroy another player’s money is significantly different under the “positive”, “negative” and “neutral” emotional state is rejected at the 10% level.” → The sentence seems to be wrongly stated, better: "The hypothesis of no difference across treatments cannot be rejected at 10%."

We apologize for this wrongly written sentence, and corrected as you suggested.

- Page 18: the p=0.0584 does not match with the one in the table.

Corrected

- Page 19: "an increase of expectation (i.e. one extra partner out of ten) increases the probability (between 0 and 1) to adopt destructive behavior with 0.045." → You mean "a unitary increase in expectation". So, it means that a 10% increase in expectation leads to a 4.5% increase in destruction? right? Maybe it would be more conventional to state it as an elasticity (thus, for a 1% increase). Moreover, can you interpret this? Is it rather inelastic? In a similar vein, check the next sentence as well.

Thank you very much for this interesting suggestion. We present results in terms of percentages.

- Page 21, top: “Both results are in line with Hypothesis 1.” → Why don't you mention that

"Expected partners’ beliefs" is not significant, which contradicts H1? Apparently, you drop this

variable in table 6b but you should explain why. Besides, table 6b is not really introduced in the text. The transition between table 6a and 6b is a bit abrupt.

We now introduce explanatory step by step and results seem to be highly consistent. We also mention the non significance of the variable “Expected partners’ beliefs”.

- Page 22:

o One more time, why using a Tobit regression? You could justify it by specifying the number

of left/right censored observations.

Thank you again for pointing this out. We justified accordingly.

o Typo : “and then regreSS first order beliefs on the second order beliefs (Table 7). To do so,

we use censored Tobit regressions...”

Corrected

- Page 23: "we should test if there exists an indirect effect of expectations about others’ beliefs on destruction" → the end of the paragraph is strange. You seem to say that you are now going to show that there is an indirect effect, but this is what you just showed in table 7. So, instead of writing “we should test if there exists an indirect effect of expectations about others’ beliefs on destruction. As expected, we found an affirmative response to this assumption.”, I would simply write “This shows that there exists an indirect effect of expectations about others’ beliefs on destruction.”.

We agree and corrected as suggested.

- Page 25:

o "Indeed, experienced participants are closer to real behaviors. " → I think that this is debatable. On the contrary, one might argue that experienced participants have learnt specific behavior that may bias their decisions. For example, they may be used to detect the goal of the experiment. In the positive emotion treatment, they may suspect that the experimenter expects subjects to destroy less, and so be influenced by this conjecture(?)

We thank you for pointing this issue which is indeed a plausible explanation. We included this argument into the manuscript.

o Typo: “and expect that partners belieVE them to be less destructive.”

Corrected

o I don’t understand your interpretation regarding the effect of risk aversion. Besides, at the

end of the day, even though you collect ambiguity aversion, you never include it in the regressions (you only focus on risk aversion).

We now also include a variable for ambiguity aversion. However neither of the variables are significant.

o Another global remark on the regressions: you do not consider arousal in the regressions. Is this because the effects are too small? You can simply state it before presenting the regressions.

We don’t use valence or arousal in the regressions. Both variables are used to control for emotion induction (section 5.1)

Discussion & conclusion

- Page 26:

o Typo: “we deduce that individuals in whom positive emotions were induced TO destroy less than others. In other words,”

We corrected.

**Reviewer #2:**

We would like to thank you again for your careful reading, interesting suggestions and support in helping us to improve our paper. For convenience, we reproduce each of your comments below, followed by our responses in blue.

1.Authors have corrected the inconsistency between “reduce” and “destroy” in the text, but I am not completely sure about the term used in the experiment given that the questions that appeared in the original submissions seemed to be a copy paste from the experiment. Do you authors screen shots of the experiment?

We provide a screenshot of the experiment (in French)

2.Authors use dummies to control for risk and ambiguity aversion. Explain what is considered by authors to be a risk averse and ambiguity averse subject.

We included some explanations considering the two dummy variables (risk and ambiguity aversion). We consider risk-averse subjects to prefer a certain payoff to a lottery with known probabilities and ambiguity-averse subjects to avoid options whose outcome probabilities are unknown (Ellsberg, 1961).

3.In table 4, authors state that “destruction is lower in the positive emotion treatment as compared to the negative emotion treatment and the neutral emotion treatment”, but the p-value is 0.78. Then, there are not statistically significant differences between these three treatments. In this table, the differences appear regarding the expectations on partners’ destruction although at a 10% level.

Thank you for pointing this issue. We absolutely agree and modified the discussion of table 4.

4.After Table 5, authors state that “the hypothesis that the proportion of individuals who destroy another player’s money is significantly different under the “positive”, “negative” and “neutral” emotional state is rejected at the 10% level”. The specification of this sentence seems to explain a Kruskal-Wallis test. In this case, there are binary combinations in which the null hypothesis points out towards no differences between the treatments compared. I do not know in which comparison a 10% level appears. In any case, the null hypothesis is always equality of medians, not differences. The result’s explanation is right, but not the previous argument.

We apologize for this wrongly written sentence, and corrected as you suggested.

5.Authors state that “Even if we cannot statistically confirm the tendency that a positive mood reduces the incentive to destroy partner’s endowment, we also observe this tendency for negative emotions contrarily to our hypothesis 2. An explanation that negative emotions (compared to neutral emotions) also decrease incentives to destroy might be explained by the moral compensation phenomenon, or more precisely by moral cleansing” With the data of Table 5 we cannot argue that there are differences between negative and neutral emotions in any of the variables compared.

Again, thank you for pointing this issue. We absolutely agree and modified accordingly. We also mention in the conclusion section that the non-significance of results might also be explained by the effect size.

6.Regarding the ex-post power analysis, what are the value authors have used to calculate it? You present it at the beginning, but the ex-post power analysis needs to be performed for all the test without statistically significant differences. That is, in Table 4 for all the comparison except for the ones with statistically significant results. Moreover, at the beginning you indicate that “a sample size of 50 observations allow us to obtain sufficient power”. I imagine that is the analysis is properly performed you will need 50 observations in each group.

We apologize for not having been more precise on the way we carried out our power analysis.

We added the following sentences to the manuscript.

We ran an ex-post power analysis using the G*power software which revealed that our sample size (respectively 55, 57 and 60 observations for the three treatments) is sufficient (power=0.8) to observe an effect size of 0.4. While Cohen (1992) recommends that wherein d = 0.20 indicates a small effect, d = 0.50 indicates a medium effect and d = 0.80 indicates a large effect, Dancey & Reidy (2006) specify that small and medium effects are more willingly available when evaluating behavioral and psychological constructs as it is the case of constructs investigated herein.

7.After result 2, the sentence beginning by “Again” needs to me restructured. It difficult to understand the argument that authors try to convey. I do not see differences regarding negative emotions.

We agree with you, and modified the discussion accordingly.

8.In table 6b, coefficients are not necessary if you provide marginal effects. Then, you can substitute them and at the end on the table you must specify that values are marginal effects, standard deviations between brackets and the levels of statistically significance. Please, remove coefficients and provide marginal effects for all the variables.

Thank you for this suggestion. We changed tables accordingly and now only provide marginal effects.

9.Given that tables 6a and 6b are measuring the same all the models must be presented in one single table. The first model will have the three explanatory variables included in the reviewed version. The second model will include positive and negative emotion. The third one will include the interactions and the third one will add the control variables. In this case, the models will be adding new variable to see the stability of results. Authors have not controlled for the emotions in the first model, have deleted one main explanatory variable in model 3 and have not controlled for individual characteristics in models 3 and 4. Please, provide the raw data and STATA commands.

Thank you for this suggestion. We now include variables step by step. We also provide raw data and stata commands.

10.At the expense of the changes suggested to the models, the variable “positive emotion” does not refer only to those subjects with low Machiavellian scores. This variable refers to all the subjects in the sample, then, a positive emotions reduced the probability of engaging in destructive behavior. But positive emotions increase destruction of those scoring higher in Machiavellian. Be careful with interpretations and with the sentences about the results.

Thank you for pointing this issue. We have rewritten comments in relation to table 6.

11.Similarly to my suggestion about the models of Table 6, please run the same models adding variables step by step. Again, provide marginal effects. Please, rewrite the interpretation of positive emotions and the interaction after result 5, it is not clear.

Thank you for this suggestion. We now include variables step by step. We also rewrote the section after result 5 and illustrated results.

12.Why do not the authors include the variable about first order beliefs in Table 8?

Thank you for this suggestion. Indeed 1^st^ and 2^nd^ order beliefs are highly correlated. We now include the variable about first order beliefs in Table 8.

13.In order to check directly result 6, authors can include in Table 6 an interaction between second order beliefs and emotions.

We choose not to introduce the interaction term between 2^nd^ order beliefs and emotions in table 6. One of the reasons is the correlation between 1st and 2^nd^ order beliefs, which also cancels the significance of 2^nd^ order beliefs in the logit regression. Emotions and 2^nd^ order beliefs are both indirect effects.

14.After Table 8, there is an explanation about table 7. Please, move this explanation after Table 7. I really think that is not accurate to use the term “optimistic”. This result just points out that the fact of having participated in lab experiments previously reduces the expectation of others reducing their partner’s income.

We corrected as suggested.

15.Regarding the explanation about the gender effect, do not use the term “optimistic” and explain it again.

We use the term “optimistic” to explain our result related to the gender effect in relation with findings in the literature.

“We also observe that men have different expectations on partners’ beliefs on one’s own destruction as women: men expect partners to belief them to be less destructive. This result might be explained by findings that men are found to be more optimistic than women [14].”
